# Patient-reported outcomes after surgery for isolated radial head fractures: a systematic review

**DOI:** 10.1007/s00402-026-06245-z

**Published:** 2026-02-26

**Authors:** Narinder Kumar, Belinda Gabbe, Richard S. Page, Filip Cosic, Lorena Romero, Emma Heath, Ilana N. Ackerman

**Affiliations:** 1 School of Public Health and Preventive Medicine, Monash University, 553 St Kilda Road, Melbourne, VIC 3000 Australia; 2 Health Data Research UK, Swansea University Medical School, Swansea University, Singleton Park, UK; 3 St John of God Hospital and Barwon Health, and Deakin University, Geelong, Australia; 4 The Alfred Hospital, Melbourne, Australia

**Keywords:** Radial head fracture, internal fixation, radial head excision, radial head arthroplasty, patient-reported outcomes, systematic review

## Abstract

**Purpose:**

The optimal surgical treatment of displaced isolated radial head fractures remains unclear and patient-reported outcomes have not been comprehensively evaluated. In this systematic review, we aimed to compare patient-reported pain, function, and return to work outcomes following open reduction internal fixation (ORIF), radial head excision, and radial head arthroplasty (RHA) in patients with isolated radial head fractures.

**Methods:**

Four electronic databases were searched for the period from January 2000 to August 2023 to identify studies comparing surgical management interventions for isolated radial head fractures. Standard methods were used for title, abstract and full-text screening and data extraction, applying PRISMA 2020 guidelines. Risk of bias was assessed using standardised checklists.

**Results:**

Eleven studies were eligible for inclusion. The mean age of participants across the studies ranged from 36 to 65 years, with almost equal gender distribution across 434 participants. Ten studies showed a high risk of bias due to methodological concerns. Follow-up periods ranged from 12 to 84 months post-operatively. Across the included studies, 179 participants (41%) underwent RHA, 139 (32%) underwent ORIF and 116 (27%) underwent excision arthroplasty. Seven studies included patient-reported functional outcomes with relatively better function for ORIF and RHA than excision arthroplasty, eight studies reported patient-reported pain outcomes with similar pain scores across the groups and only one study reported a return to work outcome showing no difference between groups. Significant variation in comparator groups and outcome instruments precluded meta-analysis.

**Conclusions:**

This review demonstrates the paucity of high-quality evidence on patient-reported outcomes after surgical management of isolated radial head fractures. There is currently no evidence to indicate any surgical treatment modality is superior with regard to patient-reported outcomes, given the limited number of studies, substantial outcome measure variation and the inherent high risk of bias in existing studies.

**Supplementary Information:**

The online version contains supplementary material available at 10.1007/s00402-026-06245-z.

## Introduction

Radial head fractures are the most common injury around the elbow, contributing to almost one-third of injuries in this region [1–3]. The usual mode of injury is a fall on an outstretched hand, most commonly in people of middle age. Timely and appropriate treatment of the fracture is vital to regain full function and stability of the injured elbow and minimize persistent sequelae. Displaced radial head fractures are commonly managed by open reduction and internal fixation (ORIF), excision of the radial head, or radial head arthroplasty (RHA) where the radial head is replaced with a prosthesis [4]. Non-comminuted fractures (with or without associated ligamentous injuries) are usually managed by ORIF, targeting anatomic reduction and stable fixation. For comminuted fractures, functional outcomes after ORIF are relatively poor due to mal-union, non-union, post-traumatic arthritis, and avascular necrosis [4]. Excision of the radial head (excision arthroplasty) restores elbow joint mobility but fails to restore stability, especially in the presence of valgus instability, concomitant ligament injuries, fractures or elbow dislocation and can result in tardy ulnar nerve palsy [5, 6]. Comminuted fractures that are not amenable to ORIF or radial head excision may be managed with RHA [4, 7, 8]. Although the assessment can be subjective, the presence of more than three bony fragments and a loss of cortical continuity in any of the fragments commonly deems a radial head fracture as unreconstructable and likely to lead to negative functional and radiological outcomes [9, 10]. RHA is also used to manage other failed treatments but has been associated with its own short-term and longer-term post-operative complications [11].

Although each surgical modality has its advantages and disadvantages, the optimal treatment for displaced radial head fractures remains a matter of debate [12, 13]. There are currently no evidence-based guidelines to direct surgeon decision-making. Common concomitant injuries including elbow dislocation, coronoid fracture, and ligamentous injuries further complicate the treatment choice and outcomes [14–16]. The incidence of these concomitant injuries with radial head fractures has been reported to be variable, ranging from 10.2 to 39% [15, 17]. A significant number of these concomitant injuries require additional surgical procedures or a longer period of immobilization which can affect the outcome, making these a separate group from isolated radial head injuries [15]. While the clinical outcomes (for example, range of motion and radiographic outcomes) of ORIF, radial head excision, and RHA have been compared for radial head fractures with or without concomitant injuries, patient-reported outcomes from surgery for isolated radial head fractures have not been systematically evaluated [18–20]. Within the paradigm of patient-centred care, this information is critical for understanding surgical outcomes from the patient’s perspective. In this systematic review, we aimed to compare patient-reported pain, function, and return to work outcomes following ORIF, radial head excision and RHA in patients with isolated radial head fractures (defined as modified Mason Type II or III fractures) [21, 22].

## Materials and methods

### Design

A systematic review of the literature was conducted. The systematic review protocol was registered on the PROSPERO International Prospective Register of Systematic Reviews (registration number CRD42022321013). The review is reported according to the Preferred Reporting Items for Systematic Reviews and Meta-Analysis (PRISMA) 2020 statement (Supplementary file 1) [23].

### Search strategy

A comprehensive search strategy (Supplementary file 2) using customised search terms was developed by a specialist research librarian, with input from a multi-disciplinary team with clinical and research experience in orthopaedics and musculoskeletal injuries. We searched the following electronic databases: Ovid MEDLINE, Ovid Embase, Cochrane Central Register of Controlled Trials (CENTRAL) and CINAHL. Databases were searched from 1 January 2000 to 25 August 2022 for studies published in peer-reviewed journals in English only. The starting year of 2000 was chosen to restrict the review to studies evaluating contemporary surgical management of radial head fractures. All database searches were re-run in August 2023 prior to manuscript preparation, to identify any further published studies.

### Eligibility criteria

We included all studies that compared patient-reported outcomes and/or return to work outcomes from surgical interventions used for the management of isolated radial head fractures (Mason II and III classification) in patients aged ≥ 18 years. The lower limit of 18 years reflects attainment of skeletal maturity as the management of radial head fractures is different in children and adolescents [24]. There was no upper age limit. Studies that reported only clinical outcomes (for example, radiographic or re-operation outcomes) or clinician-assessed outcomes (for example, range of motion or clinician-assessed functional tools) were not eligible for inclusion. We excluded single case reports, case series or studies where a surgical intervention was compared to a non-operative intervention, conference abstracts, studies including patients with ipsilateral concomitant injuries, and studies with short post-operative follow up (mean follow up < 1 year) or where the timing of follow-up data collection was unclear. Grey literature, unpublished studies, and systematic reviews were not considered.

### Study selection

Two reviewers (NK, FC or INA) independently assessed all the identified studies using Covidence software (Veritas Health Innovation Ltd, Melbourne, Australia) to determine eligibility for inclusion. Initial title and abstract screening were followed by full text screening. At each review stage, any disagreements about eligibility for inclusion were resolved through consensus, including discussion with a third reviewer (INA) where required.

### Data extraction

Two reviewers (NK, FC) independently extracted data from each included study using a customised Excel template, which was piloted on the first three studies. The following data were extracted: study characteristics (including year of publication, country, study design, study setting, research aim/question), sample details (including sample size, age, gender, fracture classification), type of surgical intervention(s), and patient-reported outcomes (measure used, scoring range and direction, follow-up timing, group-level mean score and standard deviation or median score and interquartile range, where reported, and return to work outcomes). Where studies reported composite measures that comprised clinician-assessed and patient-reported outcomes, only data for the patient-reported outcome were extracted. Data extracted by each reviewer were compared to identify any inconsistencies, which were resolved through discussion where required.

### Outcomes

The primary outcome of interest was patient-reported function, assessed using any validated standardised tool. Other outcomes of interest included patient-reported pain, health-related quality of life and return to work (assessed using binary reporting or other work-related measures).

### Risk of bias assessment

Two reviewers (NK, EH) independently assessed the risk of bias for each included study using the Newcastle Ottawa scale for non-randomised observational studies [25] and the Critical Appraisal Skills Programme (CASP) checklist for randomised controlled trials [26]. Any discrepancies in risk of bias assessment were resolved through consensus.

### Data analysis and synthesis

The study characteristics, participant demographics, and patient-reported outcomes for each of the included studies are reported descriptively. Given the considerable heterogeneity in participant samples, interventions, outcome measures (noting that some studies did not report a scoring range or scoring direction for some patient-reported instruments), and the timing of follow-up assessments, the data were unable to be pooled for meta-analysis.

## Results

### Search yield and summary of included studies

**Fig. 1:**
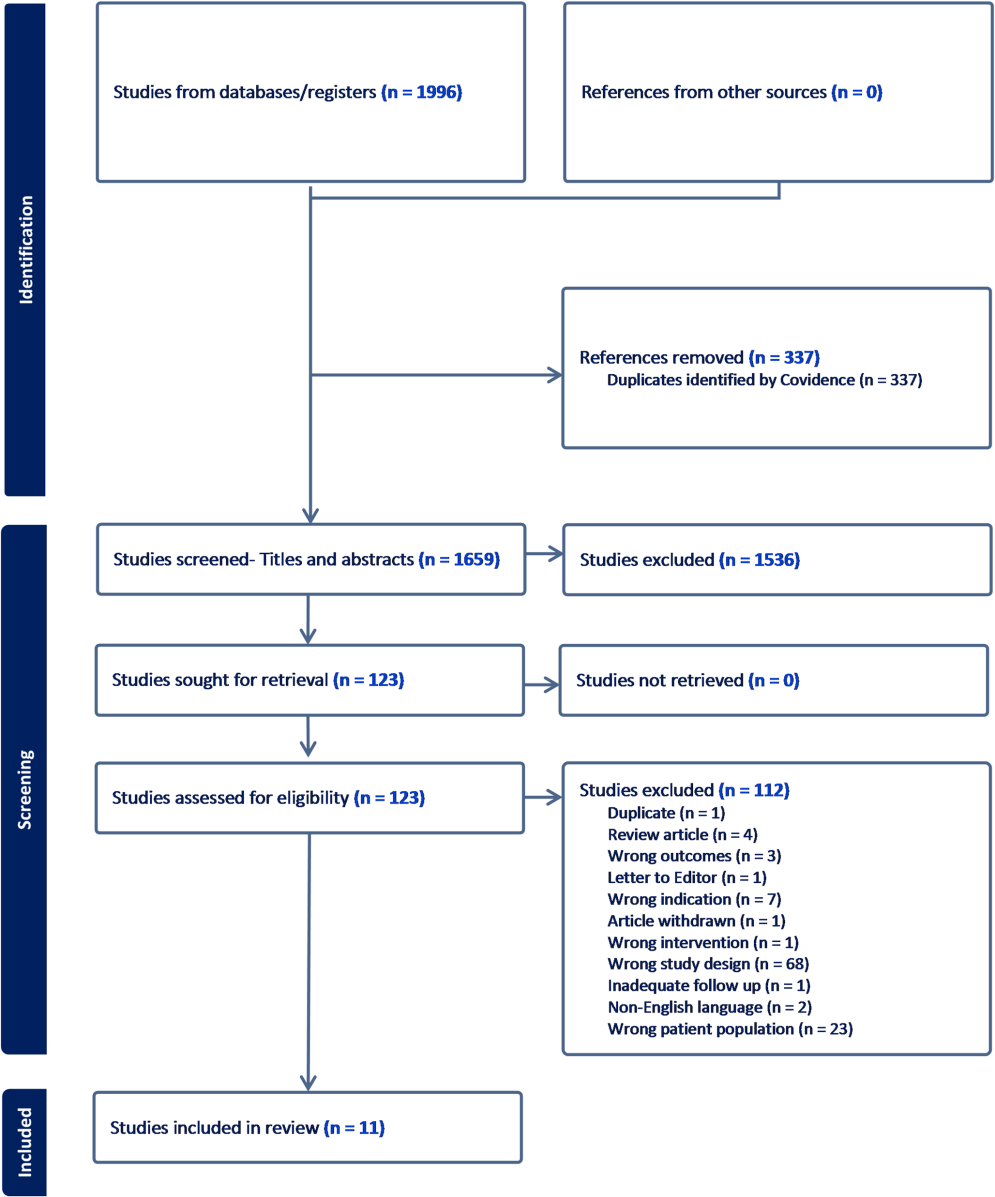
PRISMA 2020 flow diagram

The study selection and inclusion process are summarized in the PRISMA flow diagram (Fig. 1). The process of removing duplicates, screening titles, abstracts and full texts, and exclusion of studies that did not meet the eligibility criteria yielded 11 articles for inclusion in the systematic review. The included studies were from seven countries, including Turkey (*n* = 3), Italy (*n* = 2), India (*n* = 2), United Kingdom (*n* = 1), United States (*n* = 1), China (*n* = 1), and Spain (*n* = 1). The studies were published from 2010 to 2022. Of these studies, 10 were observational studies (nine retrospective cohort and one prospective cohort) and only one was specified as a randomised prospective study (assessed for the purpose of this review as a randomised controlled trial) [27].

The overall sample size in the included studies ranged from 13 to 114 participants. The mean age of study participants ranged from 36 to 64.5 years. Three studies did not report the overall mean age of participants but reported the mean age for each study group (Table 1) [28–31]. Overall gender distribution was almost equal among participants in all included studies (total of 162 males and 158 females). One study (114 participants) did not report the gender distribution of the participants [32].

**Table 1:** Study characteristics and demographics

Study characteristics				Overall sample			Excision group		ORIF group		RHA group	
Author (year)	Country (study period)	Design	Average follow-up period (range) ± SD^†^	Sample size (Mason type 2/3)	Males/females	Age (Mean and SD), [Range IQR]	n (%)	Mean age (range)	n (%)	Mean age (range)	n (%)	Mean age (range)
Akman 2017 [34]	Turkey (2009-13)	Retrospective cohort	42 months (27–72 months)	34 (0/34)	16/18	45.4 [29–64] years	15 (44.12%)	54 [38–64] years	19 (55.9%)	38.5 [29–56] years	NA	NA
Erturer 2010 [37]	Turkey (2001-08)	Retrospective cohort	Screw fixation: 32.1 months (18–63 months); K-wire: 30.5 months (11–80 months)	21 (21/0)	14/7	36 years; range 25 to 58 years	NA	NA	Screw fixation: 11 (52.4%); K-wire fixation: 10 (47.6%)	Screw fixation: 40 (30–58) years; K-wire fixation:33 (25–54) years	NA	NA
Gao 2021 [30, 31]	China (Jan 2012–Feb 2019)	Retrospective cohort	ORIF: 38.6 ± 4.5 months; Excision: 32.0 ± 6.3 months	13 (0/13)	8/5	NR	7 (53.8%)	41.7 ± 7.7 (SD) years	6 (46.15%)	37.1 ± 9.0 (SD) years	NA	NA
Gokaraju 2020 [19]	UK (5 years)	Retrospective cohort	48 months (24–81 months)	46 (12/34)	21/25	47 (range 19 to 68) years	6 (13%)	NR	28 (60.9%)	NR	12 (26.1%)	NR
Lopiz 2016* [35]	Spain (2002 -11)	Retrospective cohort	Excision: 60.3 months; RHA: 42 months	25 (0/25)	10/15	Excision: 53.7 years; RHA: 54.4 years	11 (44%)	53.7 years	NA	NA	14 (56%)	54.4 years
Meena 2017 [36]	India (Oct 2010–Jan 2013)	Prospective cohort	12 months	29 (15/14)	21/8	Excision: 44.5 ± 6.6 years; ORIF: 37.1 ± 6.2 years	15 (51.7%)	44.5 ± 6.6 years	14 (48.3%)	37.1 ± 6.2 years	NA	NA
Scoscina 2022 [28]	Italy (Jan 2014–Oct 2019)	Retrospective cohort	ORIF: 43.7 ± 11.1 months; Excision: 45.2 ± 13.2 months; RHA: 40.2 ± 8.9 months	47 (0/47)	9/38	NR	16 (34%)	64.5 ± 6.8 years	16 (34%)	41.7 ± 9.2 years	15 (32%)	53.9 ± 7.6 years
Singh 2019* [27]	India (2013–16)	Prospective randomised	20 months (18–24 months)	32 (0/32)	19/13	43 (range 22 to 70) years	15 (46.9%)	43.6 (22–70) years	NA	NA	17 (53.1%)	42.9 (23–68) years
Songy 2021 [32]	USA (15; years (2001–2015)	Retrospective cohort	6.5 years (2–16 years)	114 (NR)	50/64	53 (range 20 to 84) years	NA	NA	NA	NA	114 (100%)	53 (20–84) years
Unlu 2018 [33]	Turkey (2008–13)	Retrospective cohort	Excision: 31.1 months; RHA: 28.2 months	14 (0/14)	9/5	Excision: 42.5 years; RHA: 49 years	7 (50%)	42.5 years	NA	NA	7 (50%)	49 years
Zarattini 2012 [29]	Italy (1973–2004)	Retrospective cohort	Excision: 157 ± 61.84 months; RHA: 125 ± 39.09 months	59 (59/0)	35/24	NR	24 (41%)	41 ± 13.94 years	35 (59%)	40 ± 13.18 years	NA	NA

*NA* not applicable, *NR* not reported, *ORIF* open reduction and internal fixation, *RHA* radial head arthroplasty, *UK* United Kingdom,; *USA* United States of America

*Total of 114 participants; however, follow-up data for only 79 participants was available after 2 years

^†^Where reported

### Risk of bias assessment

Based on the Newcastle Ottawa scale, most of the observational studies were considered to be of fair or poor quality (Table 2). This was predominantly due to low scores in the “selection” domain. Only one study was rated as being of good quality (Table 2) [19]. Based on the CASP checklist, the randomised prospective study was considered to be of poor quality (Table 3).

**Table 2:** Quality assessment of cohort studies using the modified Newcastle-Ottawa scale

Author (year)	Selection				Comparability^†^	Outcome			Quality^‡^
	Representativeness of exposed cohort (⋆)	Selection of non-exposed cohort (⋆)	Ascertainment of exposure (⋆)	Demonstration that outcome of interest was not present at start of study (⋆)	(⋆⋆)	Assessment of outcome (⋆)	Was follow-up long enough for outcomes to occur?	Adequacy of follow up (⋆)	
Akman 2017 [34]	–	–	⋆	–	–	⋆	⋆	⋆	Poor
Erturer 2010 [37]	–	–	–	–	–	–	⋆	⋆	Poor
Gao 2021 [30, 31]	–	–	⋆	–	⋆	⋆	⋆	⋆	Poor
Gokaraju 2020 [19]	⋆	⋆	⋆	–	⋆	⋆	⋆	⋆	Good
Lopiz 2016 [35]	–	⋆	⋆	–	⋆	⋆	⋆	⋆	Fair
Meena 2017 [36]	–	⋆	⋆	–	⋆	⋆	⋆	⋆	Fair
Scoscina 2022 [28]	–	⋆	⋆	–	⋆	⋆	⋆	⋆	Fair
Songy 2021 [32]	–	⋆	⋆	–	–	⋆	⋆	⋆	Poor
Unlu 2018 [33]	–	–	⋆	–	⋆	⋆	⋆	⋆	Poor
Zarattini 2012 [29]	–	⋆	⋆	–	⋆	⋆	⋆	⋆	Fair

Good quality: 3 or 4 stars in selection domain AND 1 or 2 stars in comparability domain AND 2 or 3 stars in outcome/exposure domain

Fair quality: 2 stars in selection domain AND 1 or 2 stars in comparability domain AND 2 or 3 stars in outcome/exposure domain

Poor quality: 0 or 1 star in selection domain OR 0 stars in comparability domain OR 0 or 1 stars in outcome/exposure domain

^†^Comparability assessed as the following: one star awarded if the study excluded or adjusted for age, sex and marital status, another star awarded if the study adjusted for any other factor

^‡^Thresholds for converting the Newcastle-Ottawa scales to AHRQ standards (good, fair, and poor) [25]

**Table 3:** Quality assessment of randomised study using the CASP RCT checklist*

Checklist item	Yes	No	Can’t tell
1. Did the study address a clearly focused research question?	x		
2. Was the assignment of participants to interventions randomised?			x
3. Were all participants who entered the study accounted for at its conclusion?	x		
4. a. Were the participants ‘blind’ to intervention they were given?		x	
b. Were the investigators ‘blind’ to the intervention they were giving to participants?		x	
c. Were the people assessing/analysing outcome/s ‘blinded’?		x	
5. Were the study groups similar at the start of the randomised controlled trial?			x
6. Apart from the experimental intervention, did each study group receive the same level of care (that is, were they treated equally)?			x
7. Were the effects of intervention reported comprehensively?		x	
8. Was the precision of the estimate of the intervention or treatment effect reported?		x	
9. Do the benefits of the experimental intervention outweigh the harms and costs?			x
10. Can the results be applied to your local population/in your context?		x	
11. Would the experimental intervention provide greater value to the people in your care than any of the existing interventions?			x

*Singh et al. 2019 [27]

### Clinical and treatment characteristics

Participants in the included studies received three types of surgical interventions: ORIF, excision arthroplasty, or RHA (Table 1). Of the 434 participants in the included studies, 107 had a Mason type 2 radial head fracture and 213 had a Mason type 3 fracture. One study did not specifically report the Mason classification [32]. The mean follow-up period for the included studies ranged from 12 to 84 months (five studies reported follow-up times separately for each intervention group). Comparator groups varied widely between studies; seven studies reported a comparison of two interventions (ORIF versus excision in four studies; excision arthroplasty versus RHA in three studies), two studies reported comparison of all three interventions, one study reported comparison of two different types of ORIF implants, and one study reported comparison of three different types of RHA implants. Across the included studies, 139 participants (32%) underwent ORIF, 116 participants (27%) underwent excision arthroplasty, and 179 participants (41%) underwent RHA. The ORIF groups tended to be younger (range of mean age: 37.1–41.7 years) than the excision arthroplasty (range of mean age: 41.0–64.5 years) and RHA groups (range of mean age: 42.9–54.4 years).

### Patient-reported function outcomes

Patient reported function was assessed in seven of the included studies using a range of standardised instruments, as summarised in Table 4. Quick Disabilities of the Arm, Shoulder and Hand (Quick DASH) scores were reported in two studies (*n* = 93), and mean scores ranged from 14 to 28.6 for ORIF, 10–28.7 for RHA and 17–34.6 for excision arthroplasty on a 0 (best function) to 100 (worst function) scale [19, 28]. Mean DASH scores (reported in five studies, *n* = 145) ranged from 2.81 to 8.7 for ORIF, 19.91–24.8 for RHA and 3.7–25.84 for excision arthroplasty on a 0 (best function) to 100 (worst function) scale [29, 30, 33–35]. Mean Oxford Elbow scores (reported in two studies, *n* = 160) were 41 for ORIF (assessed in only one study), and ranged from 40.29 to 44.00 for RHA and from 32.43 to 38.00 for excision arthroplasty on a 0 (worst) to 100 (best) scale [19, 33]. Only one study reported the Patient-Rated Elbow Evaluation (PREE) score (*n* = 46) [19]. The mean PREE score was 12 for the ORIF group, 12 for the RHA group, and 18 for the excision arthroplasty group on a 0 (best) to 100 (worst) scale but between-group differences were not evaluated. Four studies did not report any patient-reported functional outcome. None of the included studies reported a health-related quality of life outcome.

**Table 4:** Patient-reported function outcomes

Author (year)	Quick DASH				DASH score				Oxford Elbow score			
	ORIF	RHA	Excision	*p*-value	ORIF	RHA	Excision	*p*-value	ORIF	RHA	Excision	*p*-value
Akman 2017 [34]	NR		NR		3.9 (0–15)		3.7 (0–18)	0.903	NR		NR	
Erturer 2010 [37]	NR				NR				NR			
Gao 2021 [30, 31]	NR	NR			8.7 ± 2.1		17.1 ± 4.1	0.025	NR		NR	
Gokaraju 2020 [19]	14 (SD not reported)	10 (SD not reported)	17 (SD not reported)	NR	NR	NR	NR		41 (SD not reported)	44 (SD not reported)	38 (SD not reported)	NR
Lopiz 2016 [35]		NR	NR			24.8 (0–27)	13.5 (0–21)	0.13		NR	NR	
Meena 2017 [36]	NR		NR		NR		NR		NR		NR	
Scoscina 2022 [28]	28.6 ± 13.5	28.7 ± 16.8	34.6 ± 19.5	0.76 to 0.88	NR	NR	NR		NR	NR	NR	
Singh 2019 [27]		NR	NR			NR	NR			NR	NR	
Songy 2021 [32]		NR				NR				NR		
Unlu 2018 [33]		NR	NR			19.91	25.84	0.798		40.29	32.43	0.334
Zarattini 2012 [29]	NR		NR		2.81 ± 2.73		21.82 ± 6.01	< 0.05	NR		NR	

Data reported as mean ± standard deviation or mean (range), as applicable

p-values represent between-group statistical comparisons, where reported

*NR* not reported, *ORIF* open reduction internal fixation, *RHA* radial head arthroplasty, *SD* standard deviation

### Patient-reported pain outcomes

Patient-reported pain outcomes in the included studies are summarised in Table 5. Pain was measured using a visual analogue scale (VAS) in four studies; however, the scoring range of the VAS was not mentioned in any of the studies. Three studies comparing ORIF with excision arthroplasty (*n* = 106) reported a marginally higher post-operative pain score in the excision groups, compared with the ORIF groups, although the between-group difference was only statistically significant in one study [29, 30, 34]. A further study (*n* = 46) compared all three interventions and reported similar pain scores between the groups [19]. Patient-reported pain scores were also available from the Broberg and Morrey score and from the Mayo Elbow Performance Score (MEPS), in one and two studies, respectively. Meena et al. reported marginally higher pain after excision arthroplasty, compared with ORIF, using the Broberg and Morrey pain score but the observed between-group difference was not statistically significant [36]. Both studies reporting the MEPS pain score (*n* = 57) found similar outcomes for the RHA and excision arthroplasty groups [27, 35]. Six studies did not report any pain outcomes.

**Table 5:** Patient-reported pain outcomes

Author (year)	Pain VAS				Broberg and Morrey score (pain score only)				Mayo Elbow Performance(pain score only)			
	ORIF	RHA	Excision	*p*-value	ORIF	RHA	Excision	*p*-value	ORIF	RHA	Excision	*p*-value
Akman 2017 [34]	1.5 (range 0–6)		1.8 (range 0–6)	0.696	NR		NR		NR		NR	
Erturer 2010 [37]	NR				NR				NR			
Gao 2021 [30, 31]	2.3 ± 0.8		3.0 ± 1.3	0.638	NR		NR		NR		NR	
Gokaraju 2020 [19]	1.3 (SD not reported)	1.9 (SD not reported)	2.5 (SD not reported)	NR	NR	NR	NR		NR	NR	NR	
Lopiz 2016* [35]		NR	NR			NR	NR			None- 9; Mild- 4; Moderate- 1; Severe- 0	None- 8; Mild- 3; Moderate- 0; Severe- 0	0.3
Meena 2017 [36]	NR		NR		33 ± 1.9		32.2 ± 2	0.532	NR		NR	
Scoscina 2022 [28]	NR	NR	NR		NR	NR	NR		NR	NR	NR	
Singh 2019* [27]		NR	NR			NR	NR			None- 4; Mild- 11; Moderate- 2; Severe- 0	None- 8; Mild- 6; Moderate- 1; Severe- 0	NR
Songy 2021 [32]		All groups combined: None- 48%, Mild- 42%, Moderate- 8%, Severe- 3% Subgroup scores not reported		0.2–0.8 for subgroup analysis		NR				NR		
Unlu 2018 [33]		NR	NR			NR	NR			NR	NR	
Zarattini 2012 [29]	1.20 ± 0.93		3.46 ± 2.55	< 0.01	NR		NR		NR		NR	

Data reported as mean ± standard deviation or mean (range); p-values represent between-group statistical comparisons, where reported

*NR* not reported, *ORIF* open reduction internal fixation, *RHA* radial head arthroplasty, *SD* standard deviation, *VAS* visual analogue scale

*Studies reporting number of participants with specific scores (No pain: 45, Mild pain: 30, Moderate pain: 15 points, Severe pain: 0 points)

Pain VAS instrument range and scoring direction was not explicitly mentioned in any of the studies

### Return to work outcomes

Only one study reported a return to work outcome (*n* = 21). In this study, Erturer et al. compared ORIF using screws with ORIF using K-wires [38]. The group treated with screws returned to work at a mean of 11.7 weeks (range 10–14 weeks), compared with 12.5 weeks (range 10–14 weeks) for the group treated with K-wires. There was no significant difference between the two groups for this outcome (*p* = 0.46). No other indicators of return to work, work productivity or work impairment were reported in any of the other studies.

Table 6 provides a high-level overview of the included studies.

**Table 6:** Overview of included studies

Author (year)	Key study characteristics	
Akman 2017 [34]	• Retrospective cohort (total *n* = 34) • Mean follow up: 42 months • Male: Female: 16:18	• Mean age: 45.4 years • Surgery performed: ORIF (*n* = 19) Excision (*n* = 15) • No statistically significant difference in outcomes (DASH or VAS scores) between ORIF and excision groups
Erturer 2010 [37]	• Retrospective cohort (total *n* = 21) • Mean follow up: ORIF type 1 (Screw fixation): 32.1 months ORIF type 2 (K-wire): 30.5 months • Male: Female: 14:07	• Mean age: 36 years • Surgery performed: Screw fixation (*n* = 11) K-wire fixation (*n* = 10) • No significant differences between the two fixation groups for time to return to work
Gao 2021 [30, 31]	• Retrospective cohort (total *n* = 13) • Mean follow up: ORIF: 38.6 months Excision: 32.0 months • Male: Female: 8:5	• Mean age: NR • Surgery performed: ORIF (*n* = 6); Excision (*n* = 7) • Significantly better DASH scores in ORIF group. No significant difference in pain VAS between the two groups
Gokaraju 2020 [19]	• Retrospective cohort (total *n* = 46) • Mean follow up: 48 months • Male: Female: 21:25	• Mean age: 47 years • Surgery performed: Excision (*n* = 6) ORIF (*n* = 28) RHA (*n* = 12) • Statistical significance not reported; study concluded that functional outcome was similar in all groups
Lopiz 2016 [35]	• Retrospective cohort (total *n* = 25) • Mean follow up: Excision: 60.3 months RHA: 42 months • Male: Female: 10:15	• Mean age: Excision: 53.7 years; RHA: 54.4 years • Surgery performed: Excision (*n* = 11) RHA (*n* = 14) • No significant difference in DASH scores or Mayo pain scores between groups
Meena 2017 [36]	• Prospective cohort (total *n* = 29) • Mean follow up: 12 months • Male: Female: 21:8	• Mean age: Excision: 44.5 years ORIF: 37.1 years • Surgery performed: Excision (*n* = 15); ORIF (*n* = 14) • No significant difference in pain score between groups (overall or by Mason classification)
Scoscina 2022 [28]	• Retrospective cohort (total *n* = 47) • Mean follow up: Excision: 45.2 months ORIF: 43.7 months RHA: 40.2 months • Male: Female: 9:38	• Mean age: Excision: 64.5 years ORIF: 41.7 years RHA: 53.9 years • Surgery performed: Excision (*n* = 16) ORIF (*n* = 16) RHA (*n* = 15) • No significant difference in QuickDASH scores between groups
Singh 2019 [27]	• Prospective randomised study (total *n* = 32) • Mean follow up: 20 months • Male: Female: 19:13	• Mean age: 43 years • Surgery performed: Excision (*n* = 15) RHA (*n* = 17) • Moderate-severe pain similar in both groups at 18 months
Songy 2021 [32]	• Retrospective cohort study (total *n* = 114) • Mean follow up: 6.5 years • Male: Female: 50:64	• Mean age: 53 years • Surgery performed: RHA type 1 (*n* = 60) RHA type 2 (*n* = 21) RHA type 3 (*n* = 33) • No significant difference in pain between the 3 groups
Unlu 2018 [33]	• Retrospective cohort (total *n* = 14) • Mean follow up: Excision: 31.1 months RHA: 28.2 months • Male: Female: 9:5	• Mean age: Excision: 42.5 years RHA 49 years • Surgery performed: Excision (*n* = 7) RHA (*n* = 7) • No significant differences in DASH or Oxford Elbow Scores between groups
Zarattini 2012 [29]	• Retrospective cohort (total *n* = 59) • Mean follow up: Excision: 157 months ORIF: 125 months • Male: Female: 35:24	• Mean age: Excision: 41 years ORIF: 40 years • Surgery performed: Excision (*n* = 24) ORIF (*n* = 35) • ORIF group had significantly better DASH scores and less pain than the excision group

*NR* not reported, *ORIF* open reduction and internal fixation, *RHA* radial head arthroplasty

## Discussion

This systematic review shows that there is currently a significant lack of published research on patient-reported outcomes after surgery for isolated radial head fracture. Although multiple studies and earlier reviews have reported clinical and/or radiological outcomes after radial head fracture surgery, to the best of our knowledge, this systematic review is the first one to identify, evaluate and synthesise the available evidence on patient-reported outcomes including return to work outcomes [38, 39]. The paucity of evidence (and the relatively poor quality of the available evidence, as we have identified in this review) leads to challenges in making recommendations about surgical interventions to achieve optimal patient outcomes after these prevalent fractures. Our focus on patient-reported outcomes is important and timely, given the shift towards assessing surgical outcomes through a patient-centred lens and the wide adoption of patient-reported outcome measures in other areas of orthopaedics.

In 2013, Miller et al. systematically reviewed studies evaluating the surgical treatment (RHA, ORIF and radial head resection/excision arthroplasty) of isolated Mason type 3 fractures, analysing functional outcomes and complication rates in nine included studies with a total of 195 patients [38]. Similar to our review, the included studies were quite heterogenous in their design and comparator groups, with three studies comparing ORIF with RHA or excision arthroplasty and two case series comparing all three types of interventions. In comparison, our review excluded case series (given this study design represents a very low level of evidence) and we focused on patient-reported outcomes to better understand surgical outcomes from the patients’ perspective. Moreover, this review could not recommend any surgical modality over another, similar to our review. In 2021, Lanzerath et al. systematically reviewed published literature on the treatment of isolated Mason type 2 fractures (ORIF vs. non-operative treatment) and analysed functional outcomes and complication rates in 11 included studies with a total of 319 patients [39]. The eligibility criteria for our systematic review were necessarily different, as we sought to evaluate outcomes from surgical interventions only. In addition to Mason type 2 fractures, the present review also included studies with Mason type 3 fractures but given our specific focus on patient-reported outcomes, we excluded studies that reported only clinician-assessed outcomes or other clinical or radiological outcomes. Since the outcomes measured in this review were clinical outcomes as compared to patient reported outcomes in our review, a valid comparison could not be done. In 2024, Zhao et al. published a systematic review of studies evaluating conservative versus surgical treatment of Mason type 2 radial head fractures and analysed clinical functional outcomes from four studies with a total of 271 patients [40]. However, two out of the four included studies were published in languages other than English. This review found no significant difference in clinical functional outcomes for conservative treatment, compared to surgical treatment, with fewer complications for the conservative treatment group. The findings cannot be directly compared with ours, given that our review included both Mason type 2 and 3 radial head fractures and compared outcomes from surgical modalities only and did not include conservative treatment. Additionally, our review focused on patient-reported outcomes rather than clinical functional outcomes.

Across the 11 included studies in our review, we observed that RHA was the most commonly used surgical procedure for radial head fracture (performed for 41% of study participants overall), with excision arthroplasty being the least common (performed for 27% of study participants). We note that the largest study focused on RHA [32], which contributed to the relatively high proportion of total participants who received this type of intervention and that this pattern may not reflect contemporary clinical practice. The ORIF groups tended to be younger than the excision arthroplasty and RHA groups, and this may relate to better bone quality for fracture fixation in younger patients or surgeon preference in attempting to preserve a younger joint. With respect to patient-reported functional outcomes, we found that QuickDASH scores, DASH scores, Oxford Elbow scores and PREE scores were, on average, better for patients receiving ORIF and poorer for patients receiving excision arthroplasty. Three studies comparing ORIF with excision arthroplasty reported marginally higher pain scores for the excision arthroplasty group [29, 30, 34], and one study comparing all three surgical modalities reported the highest mean pain scores in the excision arthroplasty group [19]. However, as the quality of the included studies was generally low, we maintain that there is currently insufficient evidence to identify and recommend the optimal surgical modality in terms of patient-reported pain, function or return to work outcomes.

This systematic review highlights the considerable lack of high-quality evidence on patient-reported outcomes after different forms of radial head fracture surgery. Most of the included studies were retrospective cohort studies with small sample sizes; we identified only one eligible prospective cohort study and only one randomised comparative study. There was a high risk of bias in most of the included studies, as determined using standardised tools. Concerningly, outcome measures were inconsistent across the studies and the quality of reporting of these measures was notably poor. Return to work outcomes were largely neglected. Only one study reported return to work outcomes and no studies reported any measure of work productivity or work impairment. None of the included studies assessed quality of life or health-related quality of life, which are important indicators of overall wellbeing that are widely used in contemporary orthopaedic and trauma research and clinical practice. The inconsistent follow-up time points across the studies were also problematic; standardised outcome assessment timing (for example, at 6 months or 12 months post-operatively) would facilitate future head-to-head comparisons. Furthermore, we note that there was limited geographical representation, with most of the included studies being from Europe and none from any low-income countries.

There is a clear and pressing need for well-designed prospective studies, including high-quality randomised controlled trials, that prioritise the evaluation of patient-reported outcomes from radial head fracture surgery alongside clinical or radiological outcomes. The majority of the studies included in this review were low quality designs (predominantly retrospective cohort studies) that were poorly powered and had a high risk of bias in most instances. Future studies should more rigorously evaluate patient-reported surgical outcomes by employing research approaches that appropriately account for and mitigate common sources of bias. This includes the use of representative samples and random allocation to interventions to avoid imbalances between comparison groups. The trial sample size should be large enough to enable adequately-powered statistical comparison for the primary outcomes of interest. Building the evidence base in this way (with consideration of patient-reported outcomes as well as traditional clinical outcomes) will better inform clinical teams to make appropriate treatment choices for the best possible patient outcomes. A more robust evidence base would also underpin the development of contemporary clinical guidelines for the surgical management of radial head fractures.

### Strengths

This systematic review followed established systematic review processes, in accordance with PRISMA guidelines. To synthesise the available evidence on patient-reported outcomes after radial head fracture surgery, we undertook a comprehensive search of the contemporary literature (spanning more than 22 years) across four large databases. Risk of bias and study quality were evaluated using standardised tools for the relevant study design. Two independent reviewers conducted each stage of the systematic review process (screening, study selection, quality assessment, and data extraction) with conflicts, if any, resolved through discussion and consensus. We also re-ran our database searches prior to manuscript preparation to identify any new studies potentially eligible for inclusion.

### Limitations

We also acknowledge the limitations of this review. Only studies published in English were eligible for inclusion. The reporting of isolated radial head fractures and associated injuries was based on the information reported by study authors and we note that most of the included studies did not specify the imaging technique used. We are cognisant that a magnetic resonance imaging (MRI) study showed that up to 95% of isolated fractures of the radial head may have additional injuries [41]. As none of the included studies explicitly specified the use of MRI as a pre-operative imaging modality, we acknowledge that this may have led to under-reporting of additional injuries. However, MRI assessment is still not a standard of care initial investigation for the assessment of radial head fractures. None of the studies provided any additional operative findings confirming the isolated nature of injury. It is possible that patient-reported outcomes could be worse if patients had significant additional injuries which were missed. The generalisability of the included studies is likely to be limited in view of the small sample sizes and lack of geographical breadth. While we had planned to examine factors associated with patient-reported outcomes (for example, demographic characteristics), such associations were either not assessed or were not reported in the included studies. Finally, meta-analysis was not possible due to considerable heterogeneity of the studies with respect to study samples, comparator interventions, outcome measures, and post-operative follow-up points.

## Conclusion

This systematic review highlights the current paucity of high-quality evidence on patient-reported outcomes after surgery for isolated radial head fracture. There is no strong evidence to guide optimal treatment selection for this patient population. The findings of the included studies should be interpreted with caution given the generally poor methodological quality, variability in comparator groups, and the heterogenous range of outcome measures used. Future well-designed prospective studies (including adequately powered randomised controlled trials) conducted using a standardised set of patient-reported outcome measures and post-operative time points, are urgently needed to advance the field and facilitate an evidence-based approach to the surgical treatment of these common fractures.

## Supplementary Information

Below is the link to the electronic supplementary material.

Supplementary Material 1

Supplementary Material 2
