# Supplementary material for: Patient-reported outcomes after surgery for isolated radial head fractures: a systematic review
Source: Arch Orthop Trauma Surg. 2026 Feb 26;146(1):87. doi: 10.1007/s00402-026-06245-z (PMC12946280; doi:10.1007/s00402-026-06245-z)
Supplement: Supplementary file 2 — Supplementary Material 2 [file 402_2026_6245_MOESM2_ESM.docx]

**Ovid MEDLINE**

| **#** | **Searches** |
| --- | --- |
| 1 | Radius Fractures/ |
| 2 | ((radial or radius) adj2 (head or neck)).mp. |
| 3 | 1 and 2 |
| 4 | (((radial or radius) adj2 head) and fractur*).mp. |
| 5 | 3 or 4 |
| 6 | surgical procedures, operative/ or orthopedic procedures/ or arthroplasty/ or arthroplasty, replacement/ or arthroplasty, replacement, elbow/ or fracture fixation/ or fracture fixation, internal/ or fracture fixation, intramedullary/ or open fracture reduction/ or reconstructive surgical procedures/ |
| 7 | (operating room* or operating theat* or operation room* or operation theat* or operative* or intraoperative* or perioperative* or postoperative* or surgical* or operation or peroperative* or surger* or surgeon* or postsurg* or post-surg* or excision* or resect* or reconstruct*).mp. |
| 8 | (arthroplast* or hemiarthroplast* or fracture fixation or internal fixation or intramedullary fixation or open reduction* or fracture treatment or fracture reduction or intramedullary nailing or osteosynthesis).mp. |
| 9 | 6 or 7 or 8 |
| 10 | 5 and 9 |
| 11 | (((radial or radius) adj2 head) and (arthroplast* or hemiarthroplast* or fracture fixation or internal fixation or intramedullary fixation or open reduction* or fracture treatment or fracture reduction or intramedullary nailing or osteosynthesis)).mp. |
| 12 | Radius Fractures/su and ((radial or radius) adj2 (head or neck)).mp. |
| 13 | 10 or 11 or 12 |
| 14 | limit 13 to english language |
| 15 | exp animals/ not humans.sh. |
| 16 | 14 not 15 |
| 17 | limit 16 to (case reports or clinical trial, veterinary or comment or editorial or letter or news or newspaper article or observational study, veterinary or randomized controlled trial, veterinary) |
| **18** | **16 not 17** |

**Ovid Embase**

| **#** | **Searches** |
| --- | --- |
| 1 | radius head fracture/ |
| 2 | radius fracture/ |
| 3 | ((radial or radius) adj2 (head or neck)).mp. |
| 4 | 2 and 3 |
| 5 | (((radial or radius) adj2 head) and fractur*).mp. |
| 6 | 1 or 4 or 5 |
| 7 | surgery/ or orthopedic surgery/ or surgical approach/ or surgical technique/ or fracture treatment/ or fracture fixation/ or fracture reduction/ or arthroplasty/ or elbow arthroplasty/ or replacement arthroplasty/ or resection arthroplasty/ or resurfacing arthroplasty/ or revision arthroplasty/ or intramedullary nailing/ or open fracture reduction/ or "open reduction (procedure)"/ or osteosynthesis/ or reconstructive surgery/ |
| 8 | (operating room* or operating theat* or operation room* or operation theat* or operative* or intraoperative* or perioperative* or postoperative* or surgical* or operation or peroperative* or surger* or surgeon* or postsurg* or post-surg* or excision* or resect* or reconstruct*).mp. |
| 9 | (arthroplast* or hemiarthroplast* or fracture fixation or internal fixation or intramedullary fixation or open reduction* or fracture treatment or fracture reduction or intramedullary nailing or osteosynthesis).mp. |
| 10 | 7 or 8 or 9 |
| 11 | 6 and 10 |
| 12 | (((radial or radius) adj2 head) and (arthroplast* or hemiarthroplast* or fracture fixation or internal fixation or intramedullary fixation or open reduction* or fracture treatment or fracture reduction or intramedullary nailing or osteosynthesis)).mp. |
| 13 | radius head fracture/su [Surgery] |
| 14 | 11 or 12 or 13 |
| 15 | (exp animal/ or nonhuman/ or exp invertebrate/ or animal.hw.) not exp human/ |
| 16 | 14 not 15 |
| 17 | limit 16 to english language |
| 18 | limit 17 to (conference abstract or editorial or letter or note) |
| 19 | 17 not 18 |
| 20 | case report*.mp. |
| **21** | **19 not 20** |

**EBM Reviews - Cochrane Central Register of Controlled Trials**

| **#** | **Searches** |
| --- | --- |
| 1 | Radius Fractures/ |
| 2 | ((radial or radius) adj2 (head or neck)).mp. |
| 3 | 1 and 2 |
| 4 | (((radial or radius) adj2 head) and fractur*).mp. |
| 5 | 3 or 4 |
| 6 | surgical procedures, operative/ or orthopedic procedures/ or arthroplasty/ or arthroplasty, replacement/ or arthroplasty, replacement, elbow/ or fracture fixation/ or fracture fixation, internal/ or fracture fixation, intramedullary/ or open fracture reduction/ or reconstructive surgical procedures/ |
| 7 | (operating room* or operating theat* or operation room* or operation theat* or operative* or intraoperative* or perioperative* or postoperative* or surgical* or operation or peroperative* or surger* or surgeon* or postsurg* or post-surg* or excision* or resect* or reconstruct*).mp. |
| 8 | (arthroplast* or hemiarthroplast* or fracture fixation or internal fixation or intramedullary fixation or open reduction* or fracture treatment or fracture reduction or intramedullary nailing or osteosynthesis).mp. |
| 9 | 6 or 7 or 8 |
| 10 | 5 and 9 |
| 11 | (((radial or radius) adj2 head) and (arthroplast* or hemiarthroplast* or fracture fixation or internal fixation or intramedullary fixation or open reduction* or fracture treatment or fracture reduction or intramedullary nailing or osteosynthesis)).mp. |
| 12 | Radius Fractures/su and ((radial or radius) adj2 (head or neck)).mp. |
| 13 | 10 or 11 or 12 |
| **14** | **limit 13 to english language** |

**CINAHL**

| **#** | **Query** |
| --- | --- |
| **S16** | **S14 NOT S15**  **Limiters - English Language** |
| S15 | (case report* or editorial* or letter* or note* or news*) |
| S14 | S10 OR S11 OR S12  Limiters - English Language |
| S13 | S10 OR S11 OR S12 |
| S12 | (MH "Radius Fractures/SU") AND ( ((radial or radius) N1 (head or neck)) ) |
| S11 | ( ((radial or radius) N1 head) ) AND ( (arthroplast* or hemiarthroplast* or "fracture fixation" or "internal fixation" or "intramedullary fixation" or "open reduction*" or "fracture treatment" or "fracture reduction" or "intramedullary nailing" or osteosynthesis) ) |
| S10 | S5 AND S9 |
| S9 | S6 OR S7 OR S8 |
| S8 | (arthroplast* or hemiarthroplast* or "fracture fixation" or "internal fixation" or "intramedullary fixation" or "open reduction*" or "fracture treatment" or "fracture reduction" or "intramedullary nailing" or osteosynthesis) |
| S7 | ("operating room*" or "operating theat*" or "operation room*" or "operation theat*" or operative* or intraoperative* or perioperative* or postoperative* or surgical* or operation or peroperative* or surger* or surgeon* or postsurg* or "post-surg*" or excision* or resect* or reconstruct*) |
| S6 | (MH "Surgery, Operative") OR (MH "Orthopedic Surgery") OR (MH "Arthroplasty") OR (MH "Arthroplasty, Replacement") OR (MH "Arthroplasty, Replacement, Elbow") OR (MH "Hemiarthroplasty") OR (MH "Fracture Fixation") OR (MH "Open Fracture Reduction") OR (MH "Open Reduction Internal Fixation") OR (MH "Surgery, Reconstructive") |
| S5 | S3 OR S4 |
| S4 | (((radial or radius) N1 head) and fractur*) |
| S3 | S1 AND S2 |
| S2 | ((radial or radius) N1 (head or neck)) |
| S1 | (MH "Radius Fractures") |
